# Inter-set stretch: A potential time-efficient strategy for enhancing skeletal muscle adaptations

**DOI:** 10.3389/fspor.2022.1035190

**Published:** 2022-11-15

**Authors:** Brad J. Schoenfeld, Henning Wackerhage, Eduardo De Souza

**Affiliations:** ^1^Department of Exercise Science and Recreation, Lehman College, Bronx, NY, United States; ^2^Department of Sport and Exercise Sciences, Technical University of Munich, Munich, Germany; ^3^Department of Health Sciences and Human Performance, The University of Tampa, Tampa, FL, United States

**Keywords:** hypertrophy, contraction, lengthening, force sensors, mechanical tension

## Abstract

Time is considered a primary barrier to exercise adherence. Therefore, developing time-efficient resistance training (RT) strategies that optimize muscular adaptations is of primary interest to practitioners. A novel approach to the problem involves combining intensive stretch protocols with RT. Conceivably, integrating stretch into the inter-set period may provide an added stimulus for muscle growth without increasing session duration. Mechanistically, stretch can regulate anabolic signaling via both active and passive force sensors. Emerging evidence indicates that both lengthening contractions against a high load as well as passive stretch can acutely activate anabolic intracellular signaling pathways involved in muscle hypertrophy. Although longitudinal research investigating the effects of stretching between RT sets is limited, some evidence suggests it may in fact enhance hypertrophic adaptations. Accordingly, the purpose of this paper is threefold: (1) to review how the active force of a muscle contraction and the force of a passive stretched are sensed; (2) to present evidence for the effectiveness of RT with inter-set stretch for muscle hypertrophy (3) to provide practical recommendations for application of inter-set stretch in program design as well as directions for future research.

## Introduction

Skeletal muscle is intricately involved in human locomotion and mobility, as well as playing an essential role in metabolic health (1). To accomplish its varied tasks, skeletal muscle has a high degree of plasticity, allowing it to readily adapt to various stimuli. Of note, skeletal muscle can hypertrophy, operationally defined as an increase in the axial cross-sectional area of a myofiber or whole muscle (2), when repeatedly subjected to external resistance. This capacity for growth has important implications in wellness, functional capacity, athletic performance and aesthetic pursuits (1, 2).

In humans, muscle hypertrophy is primarily achieved by the regimented performance of resistance training (RT), where myofibers are dynamically shortened and lengthened under load. Traditional RT programs involve performing multiple sets of a variety of exercises that target the body's major muscle groups. In this scenario, a set is performed for a given exercise and then the individual rests for a given amount of time (usually 1 to 3 mins) before performing another set. Depending on the RT goal, the amount of rest between sets can influence time-efficiency, either increasing or decreasing the length of the RT sessions duration.

Time is considered a primary barrier to exercise adherence (3, 4). Therefore, developing time-efficient RT strategies that optimize muscular adaptations is of primary interest to practitioners. Several popular strategies have been devised to achieve this objective, including the use of supersets and drop sets. However, while these methods can reduce the duration of training sessions, their efficacy for enhancing muscular gains remains equivocal (2).

A novel approach to the problem involves combining intensive stretch protocols with RT. Conceivably, integrating stretch into the inter-set period may provide an added stimulus for muscle growth without increasing session duration. Accordingly, the purpose of this paper is 3-fold: (1) to review how the active force of a muscle contraction and the force of a passive stretch are sensed; (2) to present evidence for the effectiveness of RT with inter-set stretch for muscle hypertrophy (3) to provide practical recommendations for application of inter-set stretch in program design as well as directions for future research.

## Theoretical basis for force and stretch-induced hypertrophy

The dry mass of a muscle is ≈70% protein (5); hence, the accretion of skeletal muscle tissue is largely a function of net protein balance (i.e., protein synthesis > protein breakdown). In this regard, muscles primarily hypertrophy when they synthesize more protein than they break down over a given time frame. Mechanical force, exercise-induced muscle damage, and metabolic stress are proposed candidate hypertrophy stimuli (6). Of these putative mechanisms, mechanical force is generally considered the most relevant to muscle growth. When a muscle is subjected to mechanical stimuli, the body initiates a response via a process called mechanotransduction, whereby these forces are transduced to chemical signals that regulate anabolic and catabolic processes through a variety of intracellular enzymatic pathways (7). Forces can be transduced longitudinally (from z-disk to z-disk within each myofibril) and/or laterally via a series of radially oriented elastic elements including the endomysium, epimysium and perimysium (8). For the purpose of this review, we will focus on the sensing of contraction and stretch-induced force (i.e., mechanosensing) as hypertrophy stimuli. Two types of force sensors are distinguished.

Active force sensors sense the force of a muscle contraction. Active force sensors either localize to the Z-disc that anchors actin, or to protein complexes called costameres that help to direct the force generated by contracting sarcomeres to the extracellular matrix surrounding each myofiber (6).

Passive force sensors are activated by the passive stretch of a muscle. Passive force sensors generally lie in parallel to the force generating actin and myosin and actually “go slack” during a force-generating concentric action that pulls on active force sensors (6). Passive force sensors generally experience force when muscles are lengthened (e.g., during a stretch).

What follows is an overview of candidate force sensors in skeletal muscle. We first discuss active force sensors and then consider passive force sensors.

There is some evidence of a filamin C-BAG3-mTORC1-protein synthesis pathway, which to date is the only mechanism that links the active force produced during a muscle contraction via an uninterrupted chain of events to muscle protein synthesis (MPS) (6). Filamin C is a Z-disc bound protein and potential mechanosensor that is coupled to BAG3. BAG3 not only regulates the degradation of filamin C by autophagy but is also linked to two mTORC1-activating mechanisms (6). The first of these mTORC1-activating mechanisms involves the binding of BAG3 to the mTORC1 inhibitors TSC1 and TSC2 via its so-called WW domain (the “WW” signifies the two tryptophans). Indeed, TSC1 appears to move near BAG3 after maximal eccentric exercise of a human muscle and at the same time activation-related mTOR phosphorylation increases (9). The second mTORC1-activating mechanism is that mechano-activated BAG3 activates the Hippo effector YAP via an intermediate step. Active YAP then increases the expression of the genes that encode the leucine transporter LAT1 (10). Consistent with this process, resistance exercise increases the expression of the leucine transporter LAT1 in human muscle (10). While this mechanism is far from fully characterized, it suggests that mechanical stress causes BAG3 to bind to mTOR and Hippo inhibitors, resulting in the activation of mTORC1-dependent protein synthesis and a greater uptake of leucine, which would further activate mTORC1 and the protein synthetic machinery.

Another candidate active force sensor is focal adhesion kinase (FAK). FAK is a component of costameres that convey the forces generated by the cytoskeleton and by sarcomeres out of the muscle fiber to the surrounding extracellular matrix (11). FAK is involved in IGF-1-induced muscle hypertrophy via TSC2 and mTORC1 (12). However, high load contractions of rat muscles do not appear to increase FAK Tyr397 phosphorylation (13). Thus, while FAK is a potential active force sensor capable of mTORC1 activation, there is currently no evidence of a functional role during hypertrophy-inducing contractions.

Regarding passive force sensors, titin is a candidate sensor as it has been proposed to respond to mechanical stretch (14). Titin, the largest known protein in the human body, possesses elastic properties that allow it to act as a molecular spring (14). Titin is anchored to the Z-disc and extends to the M-line (14), and lies parallel to the force-generating actin and myosin filaments. This suggests that titin should go slack during a shortening contraction but generate tension during passive stretch of a muscle (14). Titin has a force-activated kinase domain, so it possesses the ability to phosphorylate other proteins (15). However, there is no known signaling link to the master MPS regulator mTORC1 and consequently there is so far no evidence that the activation of titin by passive stretch increases MPS.

The nuclei of muscle fibers “flatten” during passive stretch of a muscle fiber (16). Research in non-muscle cells indicates that such nuclear flattening can activate the Hippo effector YAP, which drives the expression of the genes that encode the leucine LAT1 transporter (17). Although there are no experimental data on this as yet, we can speculate that passive stretch may cause nuclei to flatten, followed by YAP influx and activity, increased LAT1 expression (18), greater leucine influx, mTORC1 activation and ultimately greater MPS. This hypothesis remains to be tested.

Finally, when a membrane is stretched, mechanosensitive channels or stretch-activated channels (SAC) open and become permeable to ions such as Ca^2+^ and Na^+^ (19). The main function of stretch-activated channels is to “inform” the nervous system about mechanical stimuli such as the touch of the skin. It has been proposed that stretching of muscle fibers opens SACs, initiating an intracellular signaling cascade that induces calcium-dependent anabolic signaling via mTOR and its downstream target enzymes (20). The role of SACs in mechanostransduction has been demonstrated in multiple studies. For example, Spangenburg and McBride (21) showed that inhibition of SACs blunted activation of p70S6K, a key regulator of protein translation, after performance of eccentric actions. Moreover, Mirozev et al. (19) established a link between SAC activity and downstream anabolic signaling pathways in rat skeletal muscle during acute recovery following a period of mechanical unloading. These findings are consistent with evidence that blockage of SACs correlates with a blunted hypertrophic response to training in rodents (22). Although a mechanistic rationale for SAC-induced anabolic effects remains undetermined, possible mediators include enhanced cytoskeletal remodeling, calmodulin/calcineurin interactions, and elevated levels of heat shock protein-70 (23).

**Figure 1:**
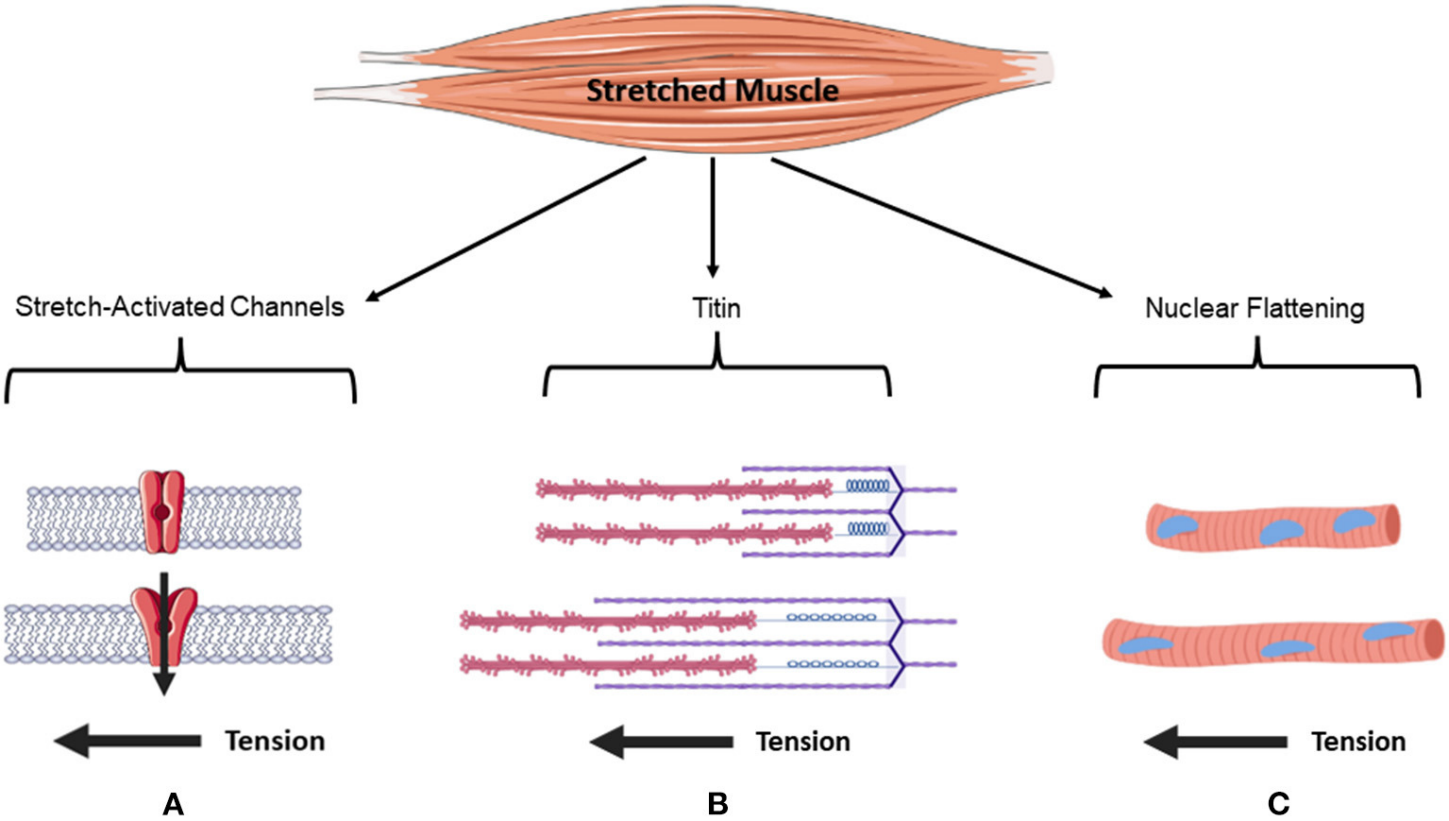
Putative mechanisms of passive sensors mediating anabolic responses.

In summary, myofibers contain active force sensors and passive force sensors. Thus, the force generated by a muscle contraction and the force that results from a passive stretch are likely sensed by different mechanosensors. While evidence is lacking for the role of passive sensors mediating anabolic responses, it is plausible to assume that their stimulation might activate mTORC1 and MPS or other events that contribute to muscle hypertrophy via several mechanisms (see Figure 1).

## The effects of passive stretch on acute anabolic factors

Given that there are several candidate force sensors that respond to passive stretch, the question arises: Can passive stretching between sets be employed as a strategy to further enhance the protein synthetic response achieved with traditional RT protocols? Emerging evidence indicates that both lengthening contractions against a high load as well as passive stretch can activate anabolic intracellular signaling pathways involved in muscle hypertrophy. In particular, an extensive body of research in rodents demonstrates increased mTOR signaling in response to passive tension. These studies include models employing stretched cultured myoblasts and myotubes (24–26) as well as *ex vivo* stretch of isolated muscles (27–29).

Evidence strongly implicates mitogen-activated protein kinase (MAPK) as an upstream mediator of stretch-induced intracellular signaling. Increased phosphorylation of MAPK has consistently been observed following passive stretch, although the degrees of involvement of specific kinases in the MAPK family (i.e., ERK 1/2, p38 and JNK) have been inconsistent (30–33). Martineau and Gardiner (32) demonstrated that p54-JNK signaling was responsive to both peak tension and the time/tension interval following *in situ* passive stretch of the rat medial gastrocnemius; the application of a contraction stimulus to the stretched muscle amplifies JNK signaling, with the greatest effects observed following eccentric actions (33).

The PI3K/Akt pathway also appears to be involved in muscle stretch. *In vitro* research by Sasai et al. (26) found that primary cultures of chick skeletal myotubes subjected to passive cyclic stretching for 72 h activates the PI3K/Akt pathway, resulting in muscle hypertrophy. Similarly, Sakamoto et al. (34) demonstrated that passively stretching the incubated fast-twitch dominant extensor digitorum longus muscle for 10 min elicited a 2-fold increase in Akt activity. Intriguingly, stretch did not significantly increase Akt activation in the predominantly slow-twitch soleus muscle despite a marked phosphorylation of MAPK p38. Adding further context to the evidence, Russ (35) electrically stimulated the tibialis anterior muscles of rats *in situ* at different frequencies across different lengths. Results showed that active contractile force had a more pronounced effect on Akt phosphorylation than passive tension; however, increasing passive tension during active muscle contraction had a synergistic effect on the degree of Akt activation. These findings suggest a potential additive role for passive tension, whereby the combination of stretch and contractile forces heighten Akt signaling and thus potentially produce a greater hypertrophic response.

Other intracellular signaling molecules have been implicated with stretch protocols, as well. Wang et al. (36) found that passive stretch (15 min, 5 days a week for 2 weeks) of the gastrocnemii in mice upregulated mRNA expression of the anabolic factors p70S6K and myogenin while downregulating the catabolic factors MuRF1, MAFbx, myostatin, and 4E-BP1. Moreover, Czerwinski et al. (37) showed that IGF-I mRNA abundance of the avian patagialis muscle increased 3-fold after 11 days of load stretch of the birds' wings. Upregulation of IGF-1 mRNA expression following passive stretch by plaster cast immobilization of the lower limb muscles in a lengthened position have been reported elsewhere in rabbits (38).

While intracellular signaling activity provides insights into anabolic and catabolic processes, skeletal muscle hypertrophy is ultimately a function of protein balance, whereby the rate of MPS exceeds that of proteolysis over time. Animal models consistently show marked increases in MPS following passive stretch protocols (39–41). These trials demonstrate that tension alone, in the absence of contractile activity, can stimulate a protein synthetic response. A notable exception to these findings by Atherton et al. (42) reported a reduction in MPS in cultured L6 skeletal muscle cells for up to 120 min after a bout of cyclic stretch. Paradoxically, results occurred despite an increased phosphorylation of intracellular signaling kinases associated with anabolism. In contrast to animal models, Fowles et al. (43) failed to demonstrate an increase in soleus MPS following ~27 min intermittent passive plantar flexor stretch in a cohort of 8 healthy men. Given the discrepancies between human and animal models, further research is required to better understand the effects of varied stretching protocols on anabolic signaling and MPS in human skeletal muscle.

## Hypertrophic adaptations to longitudinal isolated stretch protocols

Compelling evidence shows that when animals have a limb immobilized in a lengthened position it results in muscle hypertrophy while immobilizing the limb in a shortened position induces atrophy (44). A unique finding of these studies is that at least some of the hypertrophy occurs by adding sarcomeres in series (i.e., along the longitudinal axis) (44), as opposed to traditional RT protocols where a majority of hypertrophy occurs from the addition of sarcomeres in parallel (45).

Avian models consistently show marked hypertrophy when the wings of birds are stretched and then subjected to an external load (46–49). These protocols involve attaching a tube filled with lead pellets to the birds' stretched wings either on a continuous or intermittent basis over the course of several weeks. The magnitude of hypertrophy associated with such interventions appears to be greater in type I compared to type II myofibers (46). Moreover, murine research showed that manual, passive dorsiflexion stretch performed for 15 min, 5 days per week significantly increased cross-sectional area CSA of the gastrocnemii across a 2-week study period (36).

Although animal research provides interesting insights into the role of passive tension in hypertrophic adaptations, the extreme protocols employed in these models cannot necessarily be generalized to ecologically valid human stretch training. Indeed, longitudinal passive stretch interventions in humans generally do not show appreciable increases in muscle mass when performed using traditional protocols. For example, Sato et al. (50) subjected healthy males to 360 s static stretch of the plantar flexors per week for 6 weeks. Participants were randomized to perform the stretch protocol either as one weekly session or to distribute the stretch training across three sessions; total time under stretch was equated between groups. Results showed no post-study change in muscle thickness measures of the medial gastrocnemius; stretch frequency had no effect on the response. A follow-up study from the same lab reported no appreciable changes in plantar flexor hypertrophy in healthy young men after 6 weeks of two weekly 30-min stretch training sessions (51). Moreover, Junior et al. (52) reported that performing two, 25-s sets of passive stretch immediately prior to leg extension exercise attenuated muscle attenuated increases in vastus lateralis CSA compared to a RT-only condition (12.7 vs. 7.4%, respectively) over a 10-week study period. This finding was associated with a decrease in the number of repetitions performed, suggesting that negative effects of stretching prior to RT may be related to reductions in volume load (i.e., repetitions x load).

Alternatively, some evidence does show a hypertrophic benefit to passive stretch protocols. Simpson et al. (53) found that loaded stretch of the plantar flexors (performed on a leg press machine) increased gastrocnemius muscle thickness vs. a non-stretched control after 3 weeks (~11% versus ~5%, respectively); however, adaptations were similar at the end of the 6-week study period (~9% for both conditions). It is curious that a non-training control would experience appreciable hypertrophy over a relatively short time frame, raising questions as to the validity of these findings. Recently, Warneke et al. (54) reported robust increases in muscle thickness (~15%) of the lateral gastrocnemius following a 6-week stretch protocol for the calf muscles using a specially designed orthosis. It should be noted that participants stretched for an hour every day over the study period with an individual rating of discomfort of 8 on a scale of 1 to 10. Although the study provides evidence that passive stretch training can in fact promote hypertrophy in free-living humans, the high volume, frequency and intensity required to achieve results would be impractical for the majority of the population.

## Hypertrophic adaptations to inter-set stretch

Theoretically, combining passive stretch within a RT routine may enhance hypertrophic adaptations compared to RT alone. While several practical options exist, interspersing rest within the inter-set rest periods may be the best way to implement such a strategy. Ideally, stretching should be performed immediately following the final eccentric action of the RT set, which may potentiate the tension imposed on the muscles via residual effects of eccentric actions (55). In this hypothetical model, active lengthening leverages titin's role as a molecular spring by increasing its stiffness, and thus its force, to a greater extent than when passively lengthened (55). Hence, stretching immediately after eccentric loading may result in greater passive tension on the muscle following cross-bridge deactivation than what would be experienced in traditional static stretching, conceivably mediated via heightened titin stiffness (55). In addition to the heightened tension in the stretch, the strategy allows for a greater time-under-tension during a given session, which has been proposed as a driving factor in hypertrophy (56).

There is limited longitudinal research investigating the effects of stretching between RT sets, but some findings suggest it may be a viable strategy to enhance hypertrophy. Silva et al. (57) provided preliminary evidence that inter-set stretch may enhance hypertrophic adaptations. Resistance-trained men performed straight-leg plantar flexion exercise either with a standard passive rest between sets or with the inclusion of a 30-s loaded intra-set stretch. The researchers reported that the intra-set stretch condition elicited a >2-fold increase in MT of the gastrocnemius compared to control. It should be noted that these findings were presented as a conference abstract but never published in a refereed journal, thus preventing scrutiny of the study's methodology.

Evangelista et al. (58) randomly assigned untrained young men to perform an 8-week total-body RT program with either a passive between-set rest interval or a 30-s inter-set stretch integrated into the 90 s rest period. The stretch was unloaded, but reportedly performed to the point of temporary discomfort. Results indicated that the inter-set stretch condition elicited superior summed increases in muscle thickness for muscles of the upper and lower limbs vs. the passive rest condition (10.5 vs. 6.7%, respectively).

In a within-subject design, Van Every et al. (59) randomized the lower limbs of untrained young men to perform plantar flexion exercises with either a 2-min passive rest period or a 20-s inter-set stretch at the same working load followed by 100 s of passive rest. After 8-weeks, results showed greater increases in muscle thickness of the soleus for the stretch condition compared to control; these results were observed despite a decrease in RT volume load (5 to 12%) in the stretch condition. Alternatively, inter-set stretch did not show an appreciable hypertrophic benefit in the gastrocnemii. Given that the soleus is a predominantly slow-twitch muscle while the gastrocnemius is a mixed-fiber type muscle (60), these findings suggest that inter-set stretch may be more effective in hypertrophy of type I muscle fibers. This is an intriguing finding, as evidence indicates that type I fibers have a diminished hypertrophic potential compared to type II fibers (61). Thus, inter-set stretch may be a unique strategy to target development of the more “stubborn” type I fibers.

Employing an eccentric-focused protocol, Nakamura et al. (62) investigated the effects of adding a 30-s inter-set stretch to a flywheel squat program performed twice weekly in untrained young men. Results showed that improvements in strength measures tended to favor stretch compared to control; however, increases in quadriceps muscle thickness were generally similar between conditions. It should be noted that the stretch condition in this study involved moving from the flywheel unit to a massage table and then assuming the designated stretch (62), whereas the aforementioned studies performed inter-set stretch immediately after each set of RT (57–59). This time lag from moving between the exercise to the table and getting properly positioned for the stretch may have diminished the residual effects of eccentric actions on the ensuing stretch, and thus impaired hypertrophic enhancements.

In contrast to other studies on the topic, Wadhi et al. (63) found similar increases in muscle thickness and strength of the pectoralis major in a group of resistance-trained men who performed an 8-week chest-oriented training protocol consisting of flat and incline bench press exercises with either a 30-s loaded intra-set stretch or a passive rest interval. Discrepancies between studies conceivably may be explained by differences in the studies' designs. Wadhi et al. (63) employed a stretch on a different exercise (cable fly) amounting to 15% of participants working load from the prior set on the bench-press or incline bench-press exercises. While the timer under stretch was comparable to other studies (e.g., 30's), it is possible that the tension provided by the cable fly machine may not have imposed a sufficient additional stimulus to promote enhanced hypertrophic responses, particularly in the sample of trained cohorts. In addition, as with the study by Nakamura et al. (62), Wadhi et al. (63) employed a transition period between bench press performance and the stretch in the cable fly machine, which may have diminished the potential residual effects of eccentric actions on the ensuing stretch. Similar to the findings of Nakamura et al. (62), the inter-set stretching did not compromise muscular strength adaptations.

## Conclusion and practical applications

In summary, emerging evidence suggests that inter-set stretch may enhance muscular adaptations compared to traditional RT programs without increasing the time spent exercising. Its effectiveness appears to be predicated on performing the stretch immediately after the final repetition of a set, which conceivably takes advantage of the residual effects of previous eccentric actions. This research should be considered somewhat preliminary and requires further study to draw stronger inferences on its implications. However, given that several studies have observed beneficial hypertrophic effects with no evidence of a detriment, the strategy would seem to have a good cost-benefit profile. Intriguingly, despite some research showing acute strength impairments subsequent to static stretching protocols (64), current research does not indicate deleterious effects across different populations on long-term strength outcomes and in some instances the strategy shows positive adaptations (59, 62).

In addition to a general need for further exploration on the topic, several questions remain to be answered as to best practice guidelines. First, while the preliminary evidence to date suggests the need to stretch to some level of discomfort to induce additive benefits to RT, no attempts have been made to quantify the degree of intensity required to optimize results. Conceivably there is a threshold for level of tension beyond which no further increases are observed in anabolic signaling. *In vitro* research indicates that a high magnitude of strain is required to maximally stimulate p70S6K (65), but it is not clear how these findings translate *in vivo*. Based on the limited current research to date, it would seem that inter-set stretch should be carried out to a discomfort level of at least an ‘8' on the rating of perceived exertion scale (range of 1 to 10). Further study is warranted with protocols employing varying levels of stretch intensity.

Second, how long should the stretch be held between sets? Research to date has employed intra-set stretch durations of 20 to 30 s; would longer durations promote a superior benefit or perhaps blunt results by negatively impacting the volume load of the RT session? Given that 20 second bouts of high-intensity inter-set stretch have been shown to enhance hypertrophy, this should be the minimum duration employed until research indicates otherwise. Further study is warranted with differing stretch durations during the intra-set rest period.

Third, are the effects of stretching between RT sets population-specific? Age, sex and training status are known to influence exercise-induced adaptations and thus conceivably may play a role in the response to the strategy. Studies to date have focused exclusively on young men. Therefore, further studies are warranted to evaluate muscular adaptations following inter-set stretch protocols in alternative populations.

Finally, does inter-set stretch induce fiber type-specific adaptations and/or is it specific to certain muscle groups? As noted, avian models of loaded stretch show greater hypertrophy in muscles comprised predominantly of type I compared to type II myofibers (46). The aforementioned study by Van Every et al. (59) found that inter-set stretch elicited greater hypertrophy in the soleus, a type I dominant muscle, compared to the gastrocnemii, a mixed-fiber muscle. Replication of these findings and further research into the mechanisms of inter-set stretch is needed to better understand this phenomenon and its potential practical implications to program design.

## Author contributions

BJS conceived of the idea for the paper. All authors contributed to the writing and revision of the paper, and approved the final draft.

## Conflict of interest

Author BJS serves on the scientific advisory board for Tonal Corporation, a manufacturer of fitness equipment. The remaining authors declare that the research was conducted in the absence of any commercial or financial relationships that could be construed as a potential conflict of interest.

## Publisher's note

All claims expressed in this article are solely those of the authors and do not necessarily represent those of their affiliated organizations, or those of the publisher, the editors and the reviewers. Any product that may be evaluated in this article, or claim that may be made by its manufacturer, is not guaranteed or endorsed by the publisher.
